# Fission Yeast Cells Undergo Nuclear Division in the Absence of Spindle Microtubules

**DOI:** 10.1371/journal.pbio.1000512

**Published:** 2010-10-12

**Authors:** Stefania Castagnetti, Snezhana Oliferenko, Paul Nurse

**Affiliations:** 1 Cancer Research UK, Cell Cycle Lab, London, United Kingdom; 2 Biochemistry Department, University of Oxford, Oxford, United Kingdom; 3 Temasek Life Sciences Laboratory, Singapore; 4 Department of Biological Sciences, National University of Singapore, Singapore; 5 Rockefeller University, New York, New York, United States of America; National Cancer Institute, United States of America

## Abstract

Mitosis in eukaryotic cells employs spindle microtubules to drive accurate chromosome segregation at cell division. Cells lacking spindle microtubules arrest in mitosis due to a spindle checkpoint that delays mitotic progression until all chromosomes have achieved stable bipolar attachment to spindle microtubules. In fission yeast, mitosis occurs within an intact nuclear membrane with the mitotic spindle elongating between the spindle pole bodies. We show here that in fission yeast interference with mitotic spindle formation delays mitosis only briefly and cells proceed to an unusual nuclear division process we term nuclear fission, during which cells perform some chromosome segregation and efficiently enter S-phase of the next cell cycle. Nuclear fission is blocked if spindle pole body maturation or sister chromatid separation cannot take place or if actin polymerization is inhibited. We suggest that this process exhibits vestiges of a primitive nuclear division process independent of spindle microtubules, possibly reflecting an evolutionary intermediate state between bacterial and Archeal chromosome segregation where the nucleoid divides without a spindle and a microtubule spindle-based eukaryotic mitosis.

## Abstract 2

Through a previously undescribed mechanism, fission yeast cells can undergo nuclear division and enter the next cell cycle, even in the absence of spindle microtubules.

## Author Summary

The process of cell division, mitosis, ensures that chromosomes are accurately segregated to generate two daughter cells, each with a complete genome. Eukaryotic cells use a microtubule-based mitotic spindle to ensure proper chromosome segregation. In the fission yeast *Schizosaccharomyces pombe*, mitosis is “closed”: that is, the nuclear envelope does not break down, and the mitotic spindle forms within the nucleus. Unexpectedly we have found that in certain circumstances division of the fission yeast nucleus and progression into the next cell cycle can take place without the mitotic spindle. We call this nuclear division process “nuclear fission” because the nucleus separates into two bodies. We show that nuclear fission requires filamentous actin and functional spindle pole bodies, which are the fission yeast equivalent of the centrosome in other organisms. We also show that nuclear fission requires sister chromatid separation and is accompanied by some level of chromosome segregation. We propose that nuclear fission is a vestige of a primitive nuclear division process and might reflect an evolutionary intermediate between the mechanism of chromosome segregation that takes place in bacteria and the microtubule-based mitosis of modern eukaryotes.

## Introduction

Mitosis is a feature of all known eukaryotic cells, essential for the generation of viable progeny. Upon entry into mitosis the duplicated centrosomes that serve as microtubule organizing centers separate and organize a bipolar array of spindle microtubules. Microtubules are essential for chromosome segregation and eukaryotic nuclear division is not known to occur in their absence. Spindle microtubules attach to kinetochores, specialized protein complexes that assemble on centromeres of each chromosome [1]. After sister chromatid cohesion is lost at anaphase, microtubules pull the sister chromatids apart to opposite poles of the spindle. A surveillance system, the spindle checkpoint, monitors mitotic progression and prevents the onset of anaphase until all chromosomes have achieved bipolar attachment and can segregate [2]. The spindle checkpoint monitors kinetochore-microtubule attachment and a single detached or misaligned kinetochore is thought to be sufficient to trigger the checkpoint delaying the onset of anaphase and cytokinesis as well as blocking chromosome replication in the following cell cycle [3]. Defects in the spindle checkpoint result in the premature onset of anaphase and lead to chromosome mis-segregation. Genetic screens aimed at the isolation of mutants hypersensitive to microtubule depolymerizing drugs have identified many components of the spindle checkpoint [4],[5] such as *mad1*, *mad2*, *mad 3*, *bub1*, and *bub3*, which are highly conserved from yeast to humans [6]–[8].

The fission yeast *Schizosaccharomyces pombe* undergoes a closed mitosis with the nuclear membrane remaining intact and a microtubule-based spindle extending within the nucleus between two spindle pole bodies (SPB), the centrosome equivalent [9]. As in other organisms, a spindle checkpoint delays mitotic progression in the presence of microtubule defects which disrupt the spindle. The extent of the mitotic delay due to spindle checkpoint activation is variable and depends on the nature of the mitotic insult. The β-tubulin cold sensitive *nda3-KM311* mutant has no spindle microtubules and blocks in pre-prophase with condensed chromosomes [10],[11]; in contrast the temperature sensitive *nda3-1828* mutant proceeds through an aberrant mitosis and cytokinesis [12]. Here we show that in fission yeast, although depolymerization of spindle microtubules activates the spindle checkpoint, it delays mitosis only for a short time, especially at high temperature. Also, unexpectedly, in the absence of spindle microtubules, cells undergo an alternative nuclear division process and proceed into the next cell cycle. This process requires SPB separation, sister chromatid separation, and actin polymerization. We suggest that this process might represent a primitive kind of eukaryotic nuclear division.

## Results

### The Length of Mitotic Delay Due to Spindle Checkpoint Activation Is Temperature Sensitive

**Figure 1:**
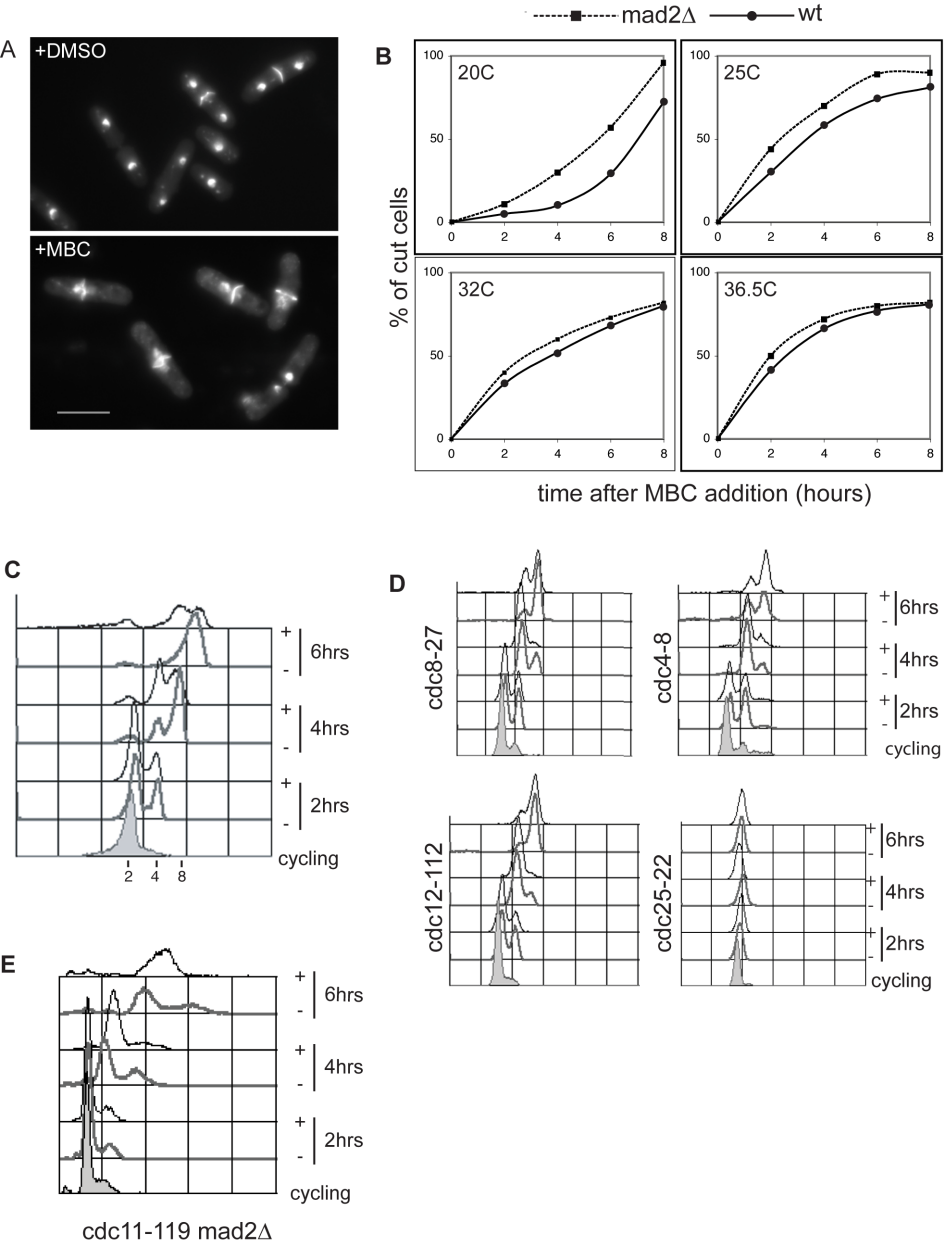
Spindle checkpoint arrest in fission yeast. (A) Dapi staining of wild type DMSO and MBC treated cells. (B) Quantification of cut cells after MBC treatment in wild type and *mad2Δ* cells. (C) Facs profile of *cdc11-119* cells before shift up (cycling) and 2, 4, and 6 h after treatment with DMSO (−) or MBC (+). (D) Facs profiles of mutants blocking cytokinesis and G2/M treated as in (C). (E) Facs profile of *cdc11-119 mad2::ura4* cells treated as in (C). Scale bar 10 µm.

We assessed the extent of mitotic and cytokinesis delay due to spindle checkpoint activation by treating wild type and spindle checkpoint deficient *mad2*Δ fission yeast cells [13] with 50 µg/ml of carbendazim (MBC), which disrupts the mitotic spindle by depolymerizing microtubules [14]. Cells that fail to segregate their chromosomes but escape the spindle checkpoint proceed through to cytokinesis and septation without completing mitosis, generating a “cut” phenotype with the septum cutting through the nucleus (Figure 1A) [15]. After MBC addition we observed a delay of cytokinesis of 2 h at 20°C and 45 min at 25°C, while at the higher temperatures of 32°C and 36.5°C no significant delays were observed (Figure 1B). These results indicate that in fission yeast MBC-dependent spindle depolymerization activates the spindle checkpoint and delays cytokinesis only transiently at low temperatures and barely at all at high temperatures. We therefore tested whether at high temperature (36.5°C) MBC treated cells could re-enter the next cell cycle and replicate their DNA. We used a temperature sensitive mutant, defective in septation initiation network signaling, which prevents cytokinesis and thus the cutting of the nucleus by the closing septum. At 36.5°C, cytokinesis defective *cdc11-119* cells treated with MBC continued DNA replication at a rate comparable to control DMSO-treated cells (Figure 1C). Similar results were obtained when additional MBC was added every hour to ensure the presence of active drug in the medium (unpublished data) and also when the cytokinesis defective mutants *cdc4-8*, *cdc8-27*, *cdc12-112* (Figure 1D), as well as *cdc7-24* and *cdc3-124* (unpublished data) were used [16],[17]. In contrast no DNA replication was observed when *cdc25-22* mutant cells blocked in G2 were treated with MBC (Figure 1D and unpublished data) [18],[19]. We conclude that if cytokinesis and septation are blocked, MBC-treated cells can proceed into the next cell cycle and undergo DNA replication. Consistently we observed no difference in DNA replication in *cdc11-119* (Figure 1C) and *cdc11-119 mad2Δ* cells (Figure 1E) treated with MBC at the restrictive temperature.

**Figure 2:**
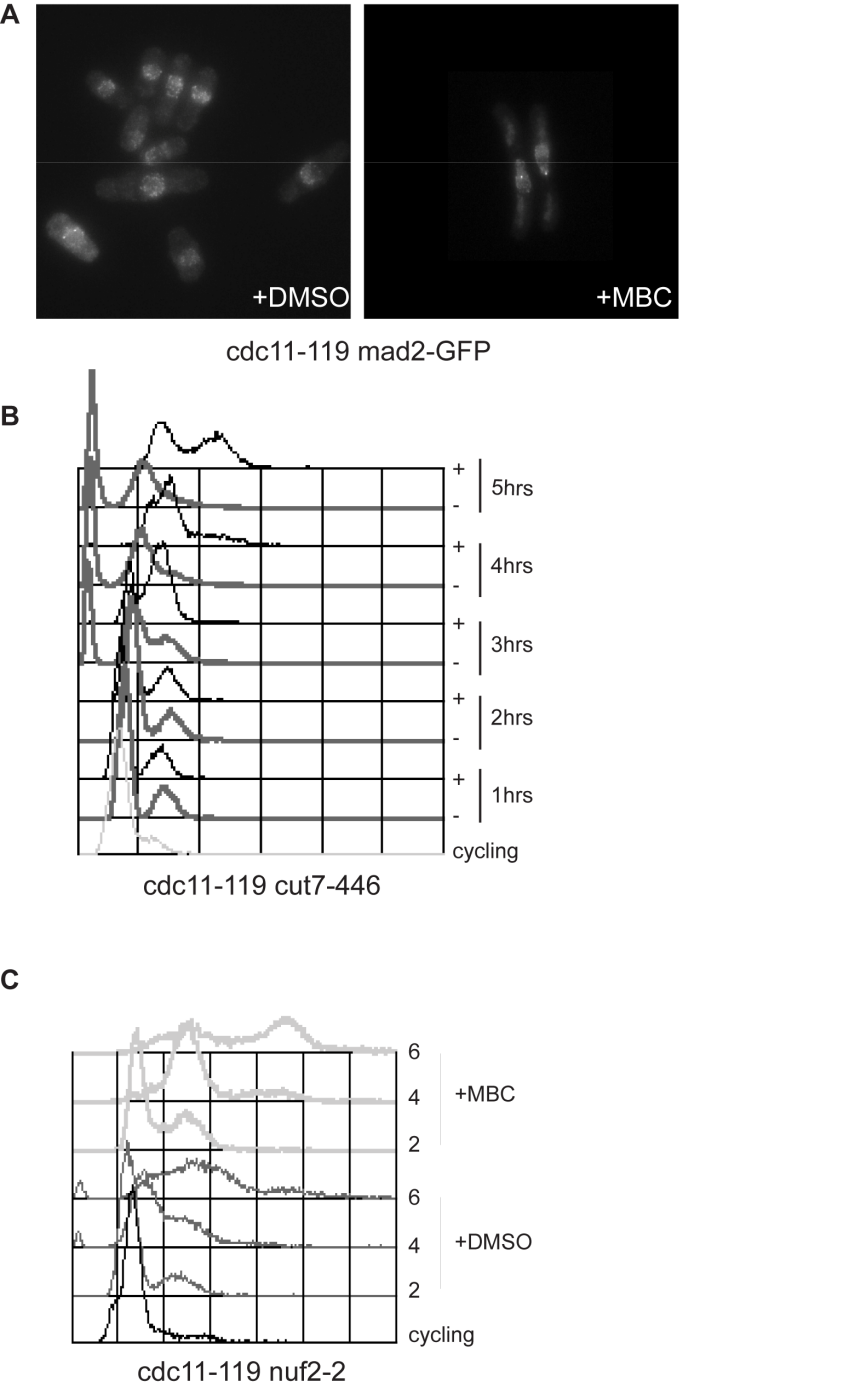
MBC-dependent microtubule depolymerization overcomes the spindle checkpoint block. (A) Mad2GFP localization in *cdc11-119* cells blocked at 36.5°C for 4 h in the presence of DMSO (left) or 50 µg/ml MBC (right). (B) Facs profiles of *cdc11-119 cut7-446* double mutant cells at 25°C (cycling) and at 36.5°C in the presence of either DMSO (−) or 50 µg/ml MBC (+). Cells were grown in EMM and nitrogen starved for 3 h prior to shift up as previously described [31]. Notably control DMSO-treated *cdc11-119 cut7-446* cells show accumulation of cut cells, despite the presence of the *cdc11-119* mutation. (C) Facs profile of *cdc11-119 nuf2-2* cells at 25°C (cycling) and at 36.5°C in the presence of either DMSO (−) or 50 µg/ml MBC (+).

The spindle checkpoint monitors kinetochore-microtubule attachment and a single unattached kinetochore is sufficient to activate the spindle checkpoint delaying the metaphase to anaphase transition and mitotic exit. We reasoned that given that MBC treated cells re-enter interphase, the spindle checkpoint may be inactive. To test this we determined whether at 36.5°C the checkpoint control was able to detect unattached kinetochores and therefore recruit checkpoint proteins such as Mad2 to the kinetochores. We investigated Mad2-GFP accumulation on kinetochores at 36.5°C and found that 21%±4% of MBC treated cells had discrete Mad2GFP loci compared to only 6%±1% in control DMSO treated cells (Figure 2A), indicating that unattached kinetochores were present and were recognized by the checkpoint machinery. Next we tested whether fission yeast cells were able to block cell cycle progression at 36.5°C in the presence of spindle microtubule defects. We used the kinesin 5-related mutant *cut7-446*, which fails to form a functional bipolar spindle due to lack of spindle microtubule interdigitation in the central region [20]. As shown in Figure 2B double mutant *cdc11-119 cut7-446* cells blocked cell cycle progression and did not replicate their DNA during the 5 h time course. MBC treatment, however, was sufficient to allow cells to proceed through the cell cycle and replicate their DNA (Figure 2B), suggesting that fission yeast cells are competent to activate the spindle checkpoint at high temperature but not in the presence of MBC. As the spindle checkpoint senses unattached kinetochores we reasoned that in MBC treated cells either the checkpoint was never activated or residual undetected microtubules bound to kinetochores inactivated the checkpoint. To distinguish between these two possibilities we utilized a strain bearing a temperature sensitive mutation in the kinetochore protein Nuf2 [21]. The *nuf2-2* allele at restrictive temperature abolishes microtubule binding, leaving the kinetochore competent to activate the spindle checkpoint [21]. As shown in Figure 2C, at their restrictive temperature *cdc11-119 nuf2-2* cells delayed mitosis and re-entered interphase more slowly than *cdc11-119* cells under the same conditions. However, when *cdc11-119 nuf2-2* cells were shifted to the restrictive temperature in the presence of MBC, DNA replication occurred more readily than in DMSO treated control cells (Figure 2C). This indicates that failure to arrest in mitosis upon MBC treatment is unlikely to be caused by the inactivation of the spindle checkpoint by residual stable kinetochore microtubules.

### Nuclear Fission in the Absence of Spindle Microtubules

**Figure 3:**
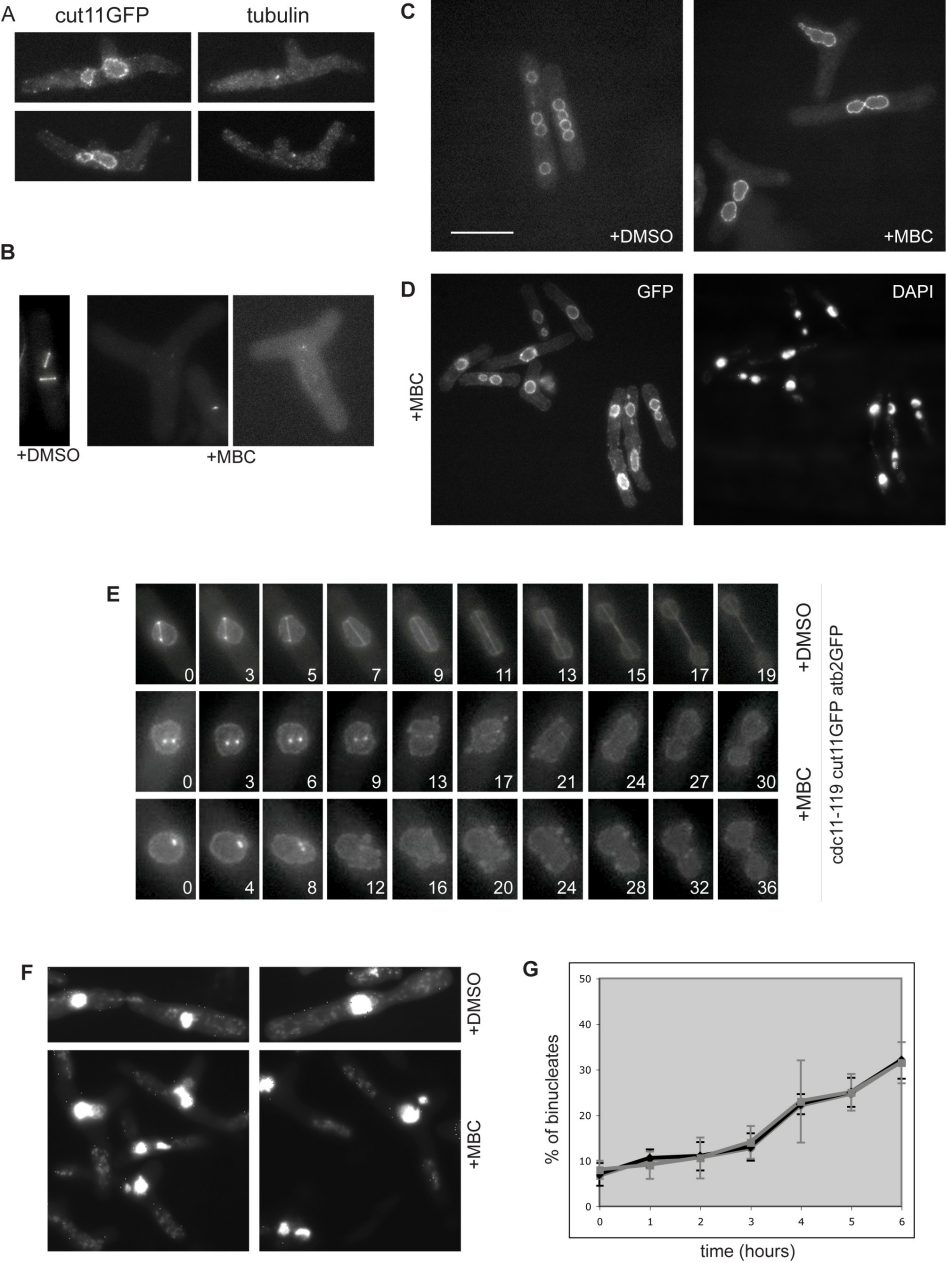
Nuclear fission in the absence of spindle microtubules. (A) Cut11-GFP and tubulin staining of MBC-treated *cdc11-119* cells (6 h at 36.5°C). (B) *cdc11-119 atb2GFP* cells at 36.5°C in MBC and DMSO. (C) Cut11-GFP staining of DMSO and MBC treated *cdc11-119 cut11-GFP* cells, 5 h at 36.5°C. (D) Cut11-GFP and dapi staining of MBC treated *cdc11-119 cut11-GFP*, after 5 h at 36.5°C. (E) Individual frames from time-lapse videos of *cdc11-119 cut11GFP atb2GFP* cells after 3 h at 36.5°C, in the presence of DMSO or 50 µg/ml MBC. (F) Dapi staining of *cut11-119 cut7-446* cells treated with DMSO or 50 µg/ml MBC. (G) Quantification of binucleated cells after MBC treatment in *cdc11-119* (black) and *cdc11-119 mad2Δ* (grey) cells. Scale bar 10 µm.

We next tested whether MBC treated cells completely lacked mitotic spindles. First, we stained for α-tubulin using antibodies and detected only very short microtubule remnants less than 1µm in length (Figure 3A), consistent with previously published data [14]. Second we could not detect mitotic spindles using a strain bearing a GFP-tagged version of α-tubulin (Atb2-GFP) (Figure 3B); only very short microtubule stubs were occasionally observed in the cytoplasm. However, despite the absence of mitotic spindle microtubules, staining with the DNA dye 4′, 6-diamidino-2-phenylindole (dapi) revealed the presence of multiple DNA masses in MBC treated *cdc11-119* cells. After 5 h at 36.5°C, 38% of the cell population had at least two well-separated DNA masses (Figure 3D). Visualization of the nuclear membrane with the marker Cut11-GFP (Figure 3A,C,D) [22] established that these DNA masses represented individual nuclear fragments enclosed by nuclear membrane. Time-lapse videos of *cdc11-119 cut11-GFP atb2GFP* cells at 36.5°C showed that the nucleus was undergoing a division process. However, unlike a normal mitosis there was no elongation of the nucleus into a dumbbell shape. Instead the nuclear envelope acquired a wobbly ruffled aspect and then eventually pinched into two nuclear masses (Figure 3E and Videos S1, S2). To confirm that the division of the nucleus was occurring without spindle microtubules, we used the double mutant *cdc11-119 cut7-446*, which fails to undergo mitosis at the restrictive temperature due to formation of monopolar spindles [20]. After 5 h at 36.5°C, 28% of the MBC treated cells had two nuclear masses (Figure 3F). Therefore, a nuclear division process takes place independently of a functional microtubule spindle. Occasional nuclear fragmentation has been reported in blocked *nda3-KM311* cells [23], and we found that after 5 h at 19°C, dapi staining of *nda3-KM311* cells showed that 30% of cells contained 2–3 nuclear bodies (Figure S1A,B). Membrane ruffling was also observed in these cells, as assayed using the nuclear envelope marker Uch2GFP [24],[25] (Video S3). We conclude that in the absence of spindle microtubules or a functional bipolar spindle, fission yeast cells can undergo an unusual nuclear division associated with ruffling of the nuclear membrane. Because the clearest characteristic of this process is fission of the nucleus, we have called it nuclear fission.

As normal mitotic progression is under surveillance of the spindle checkpoint, we tested whether this control was operative during nuclear fission. We reasoned that if nuclear fission was subject to the spindle checkpoint, then *mad2Δ* checkpoint deficient cells would undergo division more efficiently. However, we observed no difference in the accumulation of binucleates between control *cdc11-119* cells and *cdc11-119 mad2Δ* cells, suggesting that nuclear fission is independent of the spindle checkpoint control (Figure 3G and Figure S2).

### SPBs Separate in the Absence of Spindle Microtubules

**Figure 4:**
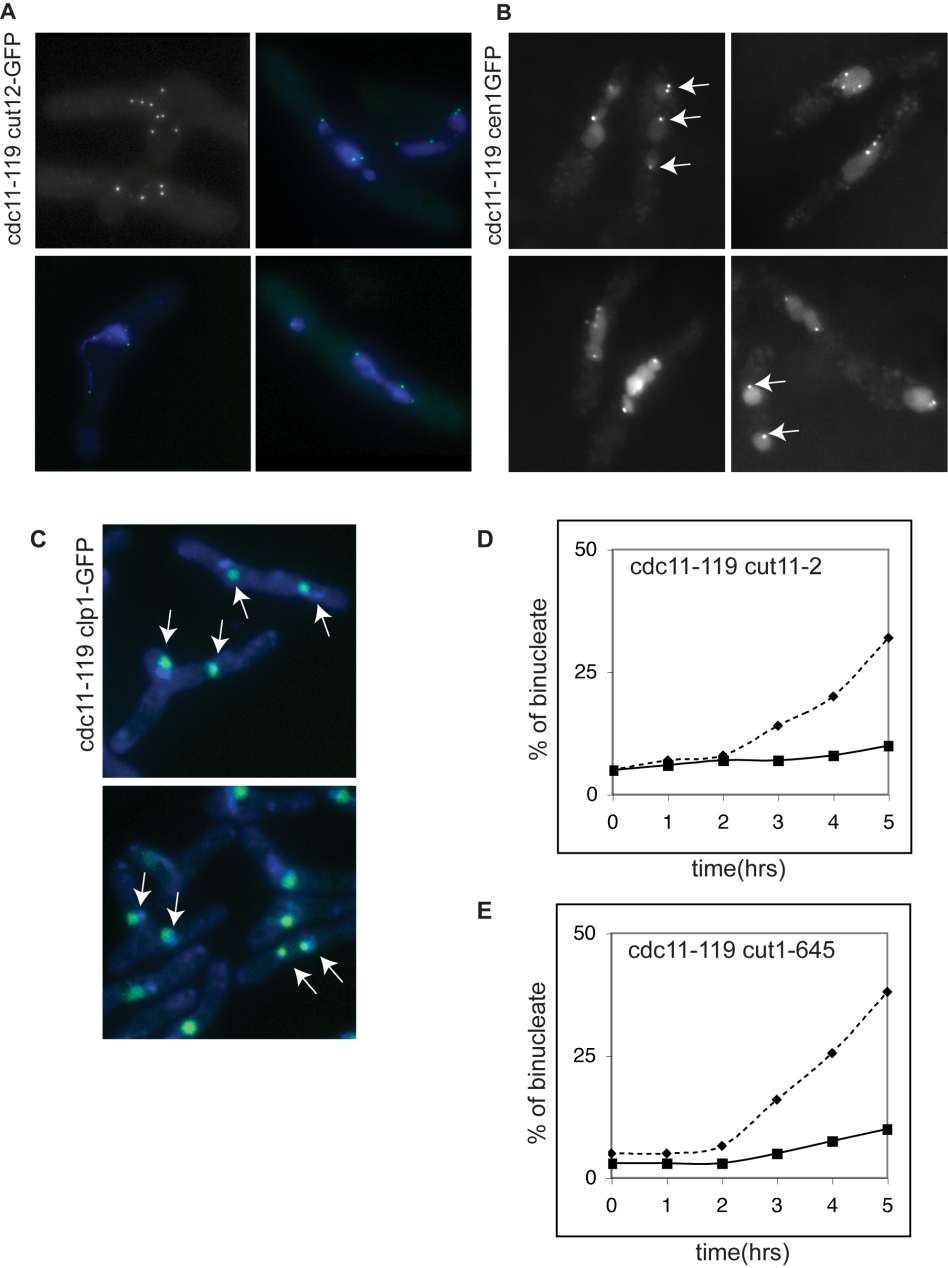
SPB and centromeres separation during nuclear fission. (A) Cut12-GFP marked SPBs in *cdc11-119* blocked cells treated with MBC for 6 h (top left) and costained with dapi (bottom panel and top right). (B) Cen1-GFP and (C) Clp1-GFP in *cdc11-119* cells. In (C) cells were co-stained with dapi. Arrows point to separated nuclear DNA masses within the same cell containing cen1GFP (B) or clp1-GFP (C). (D) Quantification of percentage of binucleated cells with two nuclear masses in *cdc11-119* (dashed), *cdc11-119 cut11-2* (black), and (E) *cdc11-119 cut1-645* (black) after MBC treatment. Scale bar 10 µm.

**Figure 5:**
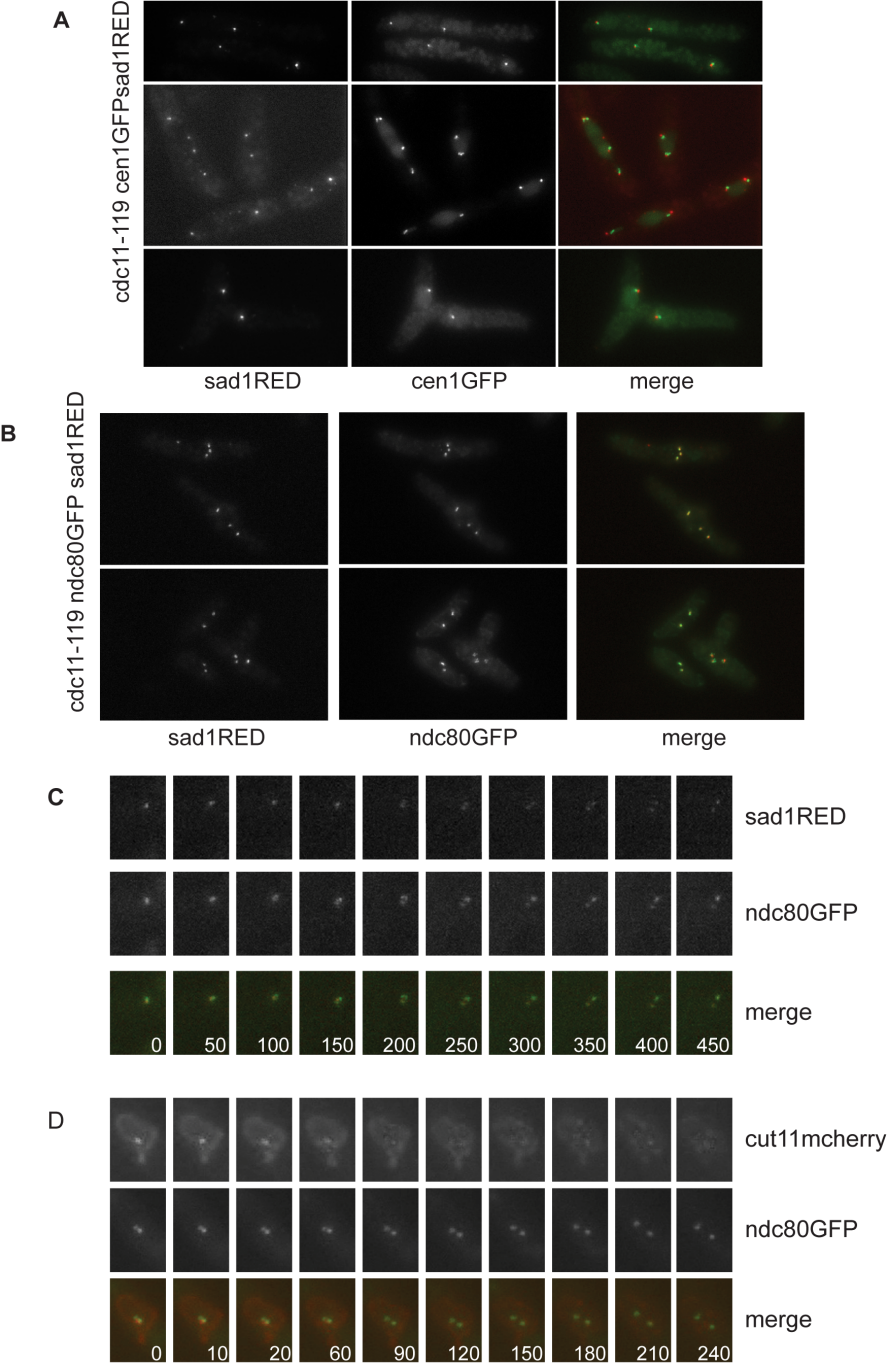
Centromeres and SPBs remain associated during nuclear fission. (A) Co-imagining of cen1-GFP and Sad1-DsRed in *cdc11-119* MBC treated cells (5 h). (B) Co-imagining of Ndc80-GFP and Sad1-DsRed in *cdc11-119* MBC treated cells (5 h). (C) Individual frames from time-lapse videos of *cdc11-119 ndc80GFP sad1DsRED* cells after 3 h at 36.5°C, in the presence of 50 µg/ml MBC. (D) Individual frames from time-lapse videos of *cdc11-119 ndc80GFP cut11mcherry* cells after 3 h at 36.5°C, in the presence of 50 µg/ml MBC. Scale bar is 10 µm.

Fission of the nuclear membrane is the final event of mitosis, so we determined whether earlier events of mitosis were also taking place during nuclear fission. At the onset of mitosis, the duplicated SPBs separate and the mitotic spindle forms between them [9],[26]. To assess whether SPB separation occurred during nuclear fission, we used two SPB markers, Cut12-GFP (Figure 4A) [27] and Sad1-DsRed (Figure 5A,B) [28]. After 5 h in MBC 82% of the *cdc11-119* cells had at least 2 SPBs (Table 1), and after 6 h cells with up to 8 SPBs were observed (Figure 4A). Significantly, almost all of the dapi-staining DNA masses (98%) were associated with at least one SPB (Figure 4A), suggesting that SPB separation was part of the process of nuclear fission. It has been previously shown that the SPBs facilitate nuclear envelope division during mitosis [25] and if SPB function is also required for nuclear fission, then impairing SPB maturation should block nuclear fission. To investigate this we monitored the appearance of binucleates in a *cut11-2* mutant that fails to anchor the SPB in the nuclear envelope and exhibits defective maturation of a new SPB [22]. As shown in Figure 4D, nuclear fission was reduced from 38%±3% in *cdc11-119* control cells to 7.5%±4% in *cdc11-119 cut11-2* cells, indicating that efficient nuclear fission, like mitosis, requires functional SPBs.

**Table 1:** Quantification of nuclear events during mitosis (+DMSO) and nuclear fission (+MBC).

Percentage of Cell With	+DMSO	+MBC
Two or more nuclear masses (cut11GFP)	100%	42%±6.8%
2 or more DNA masses (dapi)	100%	38%±4%
2 or more SPBs	98%	82%±2%
SPBs associated to separated DNA masses	100%	98%±0.5%
Separated chromosome I (cen1-GFP)	100%	100%
Segregated cen1GFP to different DNA masses	100%	73%±6.3%
2 or more nucleolar masses (clp1-GFP)	100%	70%±2%
Associated cen1-GFP and sad1-RED	100%	96%±1%

Average of 3–8 repeats each. Standard deviation was calculated and is reported in the table.

A second important event of mitosis is sister chromatid separation, which is induced by degradation of the cohesin complex component Scc1 at the metaphase-to-anaphase transition [29]. We monitored chromosome I segregation during nuclear fission using a *cen1*-GFP expressing strain to mark the centromere of chromosome I. Within 5 h, all cells showed at least 2 cen1-GFP marked dots, establishing that separation of chromosome I centromeres was taking place (Figure 4B and Table 1). Centromeres were observed to segregate to different nuclear masses in 73% of the cells, which contained two nuclear masses. Similar results were obtained for chromosome II using a cen2-GFP strain (unpublished data). To monitor chromosome III segregation we used Clp1-GFP, which marks the nucleolus and co-segregates with chromosome III [30], and found that Clp1-GFP also partitioned to different nuclear masses in 70% of the cells with two nuclear masses (Figure 4C). These results indicate that sister chromatid cohesion is lost during nuclear fission, allowing sister chromatids to move away from each other and to segregate within the different nuclear masses. If chromatid separation is required for nuclear fission, then blocking the release of sister chromatid cohesion should reduce fission efficiency. In a separase mutant (*cut1-645*) [31] sister chromatids retain some cohesion and do not separate completely. After 5 h treatment with MBC, 11.5%±2% *cdc11-119 cut1-645* cells contained two nuclear masses compared to 43%±8% in control *cdc11-119* cells (Figure 4E), demonstrating that nuclear fission is significantly reduced if chromosome separation does not take place. We conclude that during nuclear fission SPBs and sister chromatids separate in the absence of spindle microtubules, that some level of chromosome segregation can take place, and that for efficient nuclear fission functional SPBs and sister chromatid separation are required.

### Centromeres Cluster Near SPBs during Nuclear Fission

During interphase, fission yeast centromeres cluster at the nuclear envelope in the vicinity of the SPBs [32] in a microtubule independent fashion [9],[32]. This clustering is lost upon entry into mitosis when kinetochores associate with mitotic spindle microtubules [32]. We considered that kinetochores might remain associated with SPBs in the absence of mitotic spindle microtubules. We therefore monitored centromere clustering at SPBs in MBC treated cells, using a strain bearing a centromere I marked with GFP and SPB tagged with Sad1-DsRed. In contrast to a normal mitosis we did not observe the cen1GFP signal dissociating from Sad1-DsRed labeled SPBs (Figure 5A), indicating that when microtubules are depolymerized by MBC treatment SPB-centromere association persists. We confirmed that the association was maintained for all three fission yeast centromeres using an ndc80-GFP bearing strain to label all three chromosomes simultaneously. In the presence of MBC at 36.5°C, no dissociation of the Ndc80-GFP signal from Sad1-DsRed tagged SPBs was detected (Figure 5B), suggesting that centromere clustering near the SPB persists during nuclear fission. However, it was possible that centromeres transiently dissociate from the SPBs upon mitotic commitment and then are quickly recaptured. To investigate this possibility we performed time lapse imaging of SPBs and centromeres in the presence of MBC. Time-lapse imaging of *cdc11-119 sad1DsRED ndc80GFP* showed that in the presence of MBC, SPB separation occurred without centromeres declustering (Figure 5C and Video S4). The two SPBs appeared to move apart from each other with their associated set of centromeres. As declustering occurs upon mitotic commitment, we further analyzed kinetochore clustering in a *cdc11-119 cut11mcherry ndc80GFP* strain. Cut11mcherry accumulates on SPBs from early mitosis through to the metaphase to anaphase transition, and therefore acts as a marker of mitotic commitment. We observed that MBC treated, early mitotic cells (as defined by SPB-Cut11mcherry accumulation) contained Ndc80GFP labeled dots that remained in close proximity to the SPB (Figure 5D and Video S5). The Ndc80GFP labeled dots moved away from each other only after Cut11GFP came off the SPB (Figure 5D 90″). Thus, during nuclear fission, unlike mitosis, centromeres remained clustered around the SPB. If centromere-SPB association is important for nuclear fission, then a mutant, which fails to maintain clustering of kinetochores at the SPBs, should impair nuclear fission. We used an *ima1Δ* strain, which disrupts kinetochore clustering at SPBs in 75% of cells [33]. We observed that after 6 h at the restrictive temperature 14%±2% of *cdc11-119 ima1Δ* MBC treated cells underwent nuclear fission compared with 34%±4% in control *cdc11-119* cells. MBC treated *cdc11-119 ima1Δ* cells showed no significant change in nuclear ruffling compared to *cdc11-119* cells (Video S6). Thus, failure to maintain the association between centromeres and SPBs significantly reduces the efficiency of nuclear fission.

### Nuclear Fission Is Actin-Dependent

**Figure 6:**
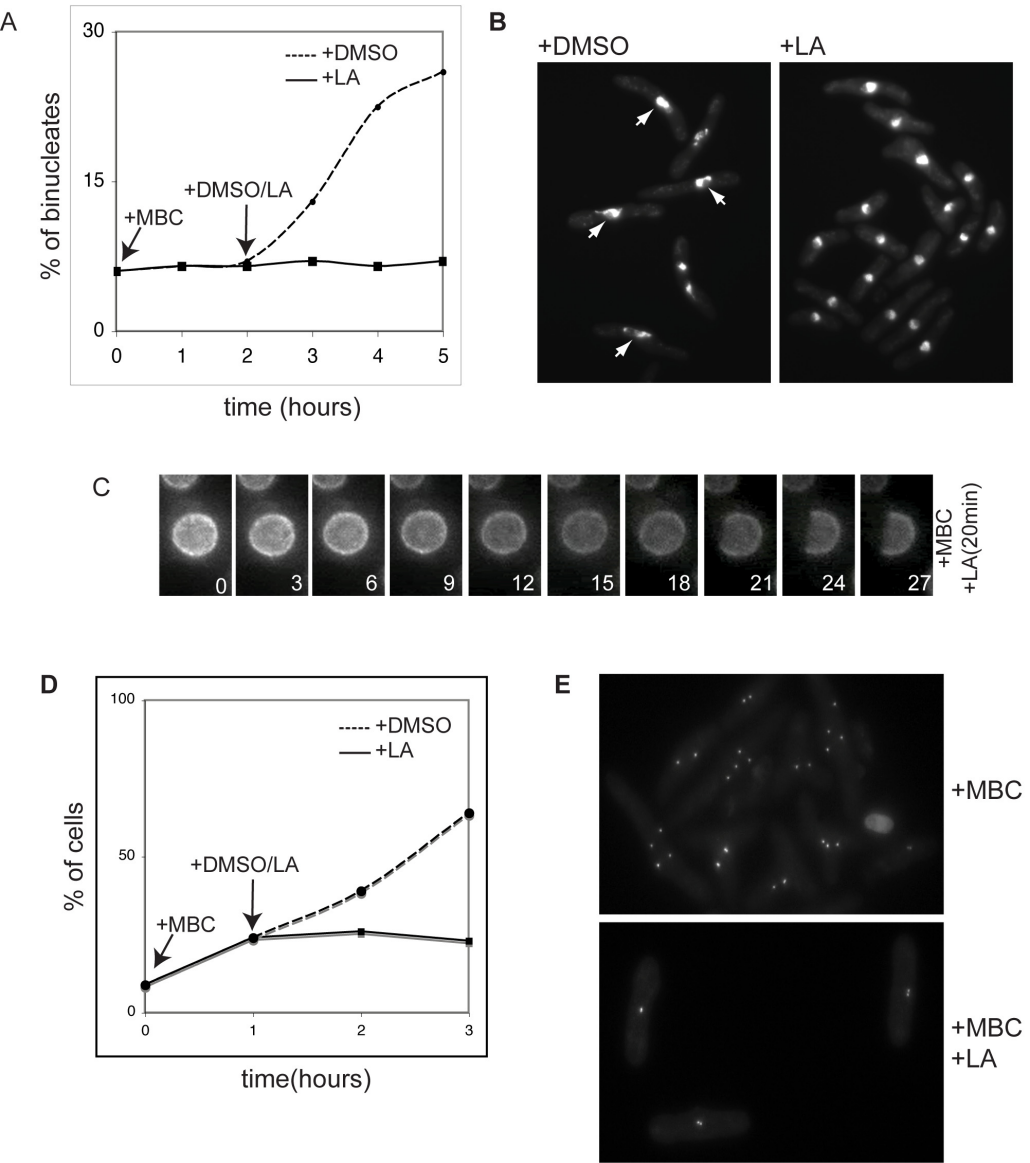
Nuclear fission and nuclear ruffling are actin-dependent. (A) Quantification of percentage of binucleated cells and (B) dapi staining of control and LA treated *cdc11-119* cells. Arrows point to enlarged nuclei that are not round in MBC treated cells; these are absent in the presence of LA. (C) Individual frames from time-lapse videos of *cdc11-119 cut11GFP* cells treated for 2 h at 36.5°C, in the presence of 50 µg/ml MBC, prior to addition of 10 µM LA. Filming was started 5 min after LA addition. At time 21 min, cut11GFP starts to be lost from the nuclear envelope and this loss continues until reaching approximately half of the nucleus. (D) Quantification of percentage of cells with 2 or more SPB in control and LA treated cells. MBC was added upon shift up to 36.5°C, and DMSO/LA was added an hour later. (E) Cut12GFP labeled SPB in LA treated *cdc11-119* cells. Scale bar is 10 µm.

Given that there are no microtubules present to generate the force necessary for nuclear fission, we ascertained whether nuclear fission required filamentous actin. *Cdc11-119* cells were treated with MBC for 2 h, followed by addition of either DMSO or 10 µM latrunculin A (LA), an actin depolymerizing drug. In MBC treated cells, we observed that LA treatment completely blocked nuclear fission (Figure 6A). Nuclei of LA treated cells were rounder than those of control DMSO treated cells (Figure 6B) and time-lapse videos of *cdc11-119 cut11GFP* cells showed no nuclear membrane ruffling (Figure 6C and Video S7). Mitosis proceeded normally in the presence of 10 µM LA when MBC was not present. As in *cdc11-119* cells, LA treatment blocked nuclear fission in *nda3-KM311* cells at 19°C (Figure S1C). Despite the dependency of nuclear fission on the actin cytoskeleton we were unable to detect actin structures in or around the nucleus (Figure S3).

We next examined whether SPB separation was also affected in LA treated cells, by blocking cells expressing the SPB marker Sad1-RED for 1 h in the presence of MBC and then treating them either with DMSO or 10 µM LA. After 1 h MBC treatment, 24%±3% of cells had 2 SPBs. After a further 2 h in the presence of LA, 23%±2% of cells had 2 SPBs, compared to control DMSO treated cells, which showed 64%±2% of cells with 2 or more SPBs (Figure 6D). The SPBs in LA treated cells also remained in close proximity to each other compared to control cells (Figure 6E). Similarly we observed no increase in the number of cells with 2 Ndc80-GFP labeled kinetochores in LA treated cells (unpublished data). Thus, we conclude that nuclear fission depends on filamentous actin.

## Discussion

**Figure 7:**
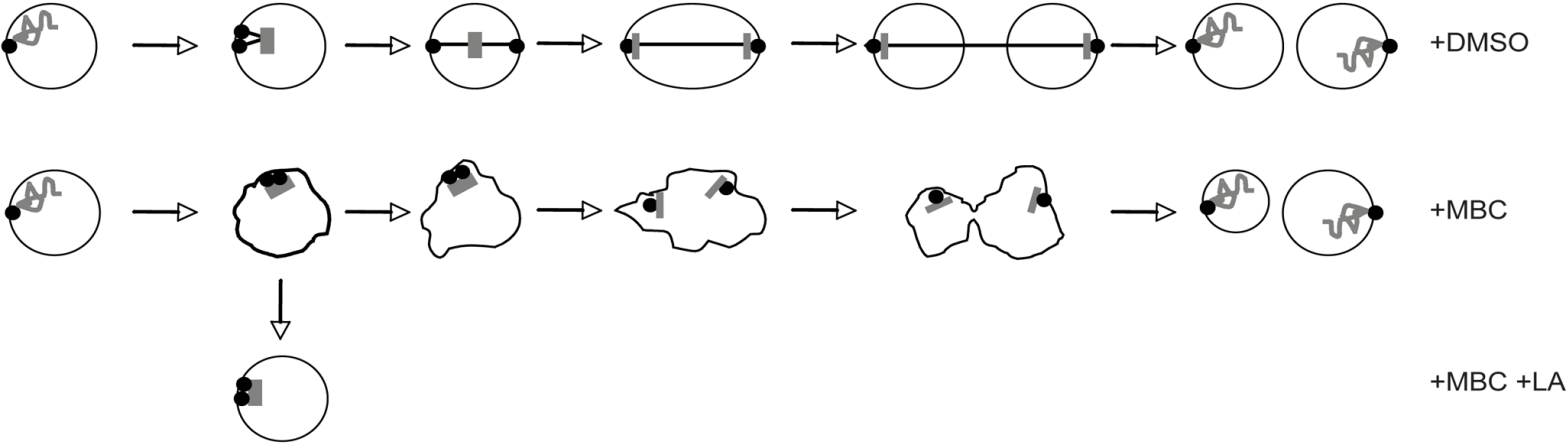
Nuclear division with and without spindle microtubules. Top panel: at the beginning of mitosis, chromosomes condense, detach from the SPB, and attach to spindle microtubules that emanate from the SPBs to form a bipolar array. Extension of the spindle and motor driven movement of chromosome along the spindle microtubules results in two daughter nuclei with equal chromosomal segregation. Lower panel: absence of spindle microtubules (+MBC), chromosomes remain associated with the SPBs and upon loss of cohesion are drugged away from each other by the movement of the SPB in the nuclear envelope. Actin depolymerization (+MBC +LA) blocks SPB separation, nuclear fission, and membrane ruffling.

This work describes an unexpected process whereby in the absence of a mitotic spindle the fission yeast nucleus can undergo nuclear division. This process, which we have called nuclear fission, requires SPB maturation and sister chromatid separation and leads to some sister chromatid segregation. We propose the following mechanism for nuclear fission (Figure 7). As cells exit G2, sister chromatids remain clustered at the SPBs. The duplicated SPBs separate slowly by moving within the nuclear membrane, and as the sister chromatids lose cohesion, they move apart as a consequence of their association with SPBs. This mechanism assumes that the two sister chromatids are associated non-randomly with different SPBs. As the chromatids separate through the nucleus, the nuclear membrane deforms around the two DNA masses generating two nuclear bodies. Preventing SPB maturation or maintaining sister chromatid cohesion interferes with the separation of DNA masses and blocks nuclear fission. Although nuclear fission occurs in the context of closed mitosis, it is reminiscent of the formation of multiple nuclei in metazoan cells when a portion of the DNA becomes separated form the bulk of the chromosomes and is encapsulated in a separate nuclear entity. This happens under pathological conditions, for example in cancer cells as a consequence of inappropriate chromosome segregation or chromosome breakage [34],[35], or during oocyte meiosis and early developmental stages in mice deficient for the chromokinesin *Kid* [36], which show incomplete chromosome compaction. It also occurs in more physiological situations such as the formation of karyomeres in the early embryonic divisions of *Xenopus*, sea urchin, and polychaetes, where individual chromosomes are separated and engulfed by the nuclear envelope [37],[38]. Important for the formation of separate nuclear entities are the necessity for a minimal distance between DNA masses and for sufficient nuclear membrane to be available. Similarly in fission yeast, nuclear fission occurs only when SPB-chromosomes masses move away from each other and when lipid biosynthesis is up-regulated during the expansion of the nuclear envelope to accommodate the elongating spindle [39]–[41]. Understanding the regulation of nuclei formation during nuclear fission might shed light on the mechanism that controls the formation of a single nucleus around each chromosome complement at the end of mitosis.

Actin polymerization, which is required for nuclear fission, might be involved in the membrane redistribution associated with the increase in nuclear envelope area observed during early anaphase B. Nuclear envelope ruffling could be a consequence of a rapid redistribution of membranes between ER and nuclear envelope. During nuclear fission, ruffling is most obvious after cut11GFP comes off the SPBs at a stage corresponding to anaphase B when spindle elongation takes place during a normal mitosis. Therefore, nuclear envelope ruffling might be expected to be more obvious during nuclear fission when there is no spindle elongation to stretch the nuclear membrane. Consistently, nuclear ruffling in fission yeast is also observed during mitosis if spindle elongation is blocked, as in the kinetochore mutant *nuf2-3* (our unpublished observation). Nuclear membrane expansion was also observed in budding yeast cells, blocked in mitosis with nocodazole to depolymerize microtubules. It has been suggested that this expansion is a consequence of the up-regulation of lipid biosynthesis normally taking place during mitosis [42]. Nuclear membrane extensions appear also upon deregulation of phospholypid biosynthesis by *spo7* inactivation [41],[42]. However, such extensions are not observed if spo7 inactivation takes place during anaphase probably because of the incorporation of extra membrane into the elongating nucleus [41]. Further studies will be required to clarify whether phospholipid biosynthesis has a role in nuclear fission or if actin is involved in the nuclear expansion observed during a normal mitosis. However, there could be other roles for the involvement of actin in nuclear fission. Actin could generate either a pushing force causing nuclear membrane distortion as is seen during lamellipodia protrusion [43] or a pulling force separating SPBs and their cargo of chromosomes. In this context it is of interest that bacterial chromosome segregation is driven by polymerization of the actin-like MreB/ParM protein [44],[45] and also that in vertebrates the driving force for centrosome separation is provided by actin filaments [46]–[48].

Differently from a normal mitosis, nuclear fission appears not to be under spindle checkpoint control and takes place irrespective of checkpoint engagement. We observed nuclear fission both in *nda3-KM311* cells, which activate the spindle checkpoint blocking cells in pre-prophase, and in MBC treated *cdc11-119* cells, which do not delay mitotic exit. Interestingly, in both circumstances cells accumulate Mad2 on kinetochores, suggesting that unattached kinetochores have been detected. However in MBC treated cells, where microtubules are almost completely depolymerized, no mitotic delay is observed and cells re-enter interphase. This result suggests that Mad2 accumulation on kinetochores, although necessary for checkpoint activation, might not be sufficient to maintain the mitotic block. Alternatively the checkpoint might be activated normally but only transiently. As a monopolar spindle does activate the checkpoint (*cut7-446*) and we have excluded the possibility that the binding of residual kinetochore microtubules inactivates the checkpoint (*nuf2-2*), something other than microtubules would have to be involved in the inactivation. It has been shown that in budding yeast activation of the APC inhibits the mitotic checkpoint through APC-mediated degradation of the checkpoint kinase Mps1 [49]. This feedback between APC and the spindle checkpoint implies that if enough APC is activated the checkpoint is turned off. In *S. pombe*, such a mutual inhibition would mean that if the nuclear fission process were to generate enough active APC, then the checkpoint block would be overcome. Alternatively, maintenance of the SPB-kinetochore interaction might cause kinetochores to be less readily available for full strength checkpoint activation, leaving sufficient levels of active APC in the nucleus to turn off the checkpoint. Further studies will be required to understand how MBC-mediated microtubule depolymerization inactivates the spindle checkpoint in fission yeast.

We speculate that nuclear fission might be a vestige of an ancient mechanism of nuclear division that predates the appearance of a mitotic spindle. It might reflect an evolutionary intermediate state between the mechanism of chromosome segregation seen in bacteria and Archea [44],[50] and that seen during mitosis in eukaryotic cells. In the intermediate evolutionary state, the replicated sister chromatids would remain attached to the centrosomes and become segregated by movement of the divided centrosomes within the nuclear membrane. Later in evolution, addition of a mitotic spindle between the centrosomes would have increased the efficiency of centrosome separation and the microtubule-based polar movement of sister chromatids would have led to a more efficient and accurate conventional mitosis. It has been observed that in the absence of the tubulin homologue FtsZ, the L-form of *Bacillus subtilis* acquires an unusual mode of proliferation with cells undergoing membrane ruffling prior to the formation of protrusions, which then resolve into independent round bodies [51]. It has been suggested that this extrusion-resolution mode could either be driven by force generated by the actin homologue MreB or by active chromosome segregation followed by collapse and resealing of the membrane. These mechanisms may be relevant to the nuclear fission process we have observed in fission yeast in the absence of spindle microtubules. A spindle independent mechanism (SIM) has also been reported for nucleolar segregation during *Aspergillus nidulans* mitosis [52]. The mechanism underlying SIM in *A. nidulans* is not yet clear, but the nuclear envelope is believed to play a critical role to generate the force necessary for nucleolar separation. In summary, we suggest that nuclear fission represents the vestiges of a primitive nuclear division process that existed early in the eukaryotic lineage prior to the evolution of a mitotic spindle and mitosis as known today.

## Materials and Methods

All *S. pombe* strains used in this study are listed in Table S1. Standard methods were used for growth and genetic manipulation [53]. All experiments, unless otherwise stated, were performed in YE4S (yeast extract with added 250 mg/l histidine, adenine, leucine, and uridine).

Cells were grown at 25°C to 1–2×10^6^ cell/ml density before shifting to the restrictive temperature (36.5°C). After 3 h (unless otherwise stated), cultures were split in two and treated with either 50 µg/ml MBC (freshly made in DMSO) or DMSO at 36.5°C, unless otherwise stated. It should be noted that some batches of MBC are more toxic for cells and these were not used in this study. For lat A treatment, following a 2 h block at 36.5°C in the presence of either 50 µg/ml MBC or DMSO, cells were treated with 12.5 µM lat A.

For immunofluorescence, cells were collected by filtration and then fixed. Cells were fixed in −80°C methanol for 1 h and then processed as previously described [54]. For microtubule detection, TAT1 antibody (a-tubulin antibody; a kind gift of Prof. K. Gull) was used at 1∶200 dilution and Alexa fluor 546-linked anti-mouse (Molecular Probes) at 1∶1000 dilution as secondary antibody.

For actin staining, cells were fixed by adding formaldehyde (final concentration 3.7%) to the medium for 25 min, washed twice with PEM (100 mM Pipes, 1 mM EGTA, 1 mM MgSO4 pH = 6.9), permeabilized with 1% Triton X-100, washed twice with PEM, and stained with rhodamine phalloidin (Molecular Probes).

For dapi staining, cells were either heat fixed (70°C) or fixed in 70% cold ethanol, then re-hydrated in distilled water, and stained with 2 µl of 50% glycerol, 0.1 M Tris pH 8 containing 1 µg/ml dapi. For immunofluorescence, cells were fixed in cold methanol at −80°C overnight and then processed as previously described [54]. Images were taken using a Deltavision microscope. The percentages are averages of 3–8 experiments and the standard errors were calculated and reported.

For live imaging cells were attached to coverslips using soya bean lectin (100 µg/ml) and imaged in minimal medium either containing DMSO or 50 µg/ml MBC at 36.5°C, using a Deltavision microscope supplied with a temperature controlled chamber.

## Supporting Information

Figure S1 **Nuclear fission in nda3KM311 cells is actin-dependent.** nda3-KM311 cells were blocked at 19°C for 3 h prior to addition of either DMSO or 10 µM LA. (A) dapi (top) and actin (bottom) staining of nda3-KM311 blocked cells treated with DMSO (left) or 10 µM LA (right). (B) Quantification of cells undergoing nuclear fission in the presence of either DMSO or LatA. (C) Mad2GFP accumulates on kinetochores of nda3-KM311 cells at the restrictive temperature in the presence of DMSO (top) or LA (bottom), indicating commitment to mitosis and spindle checkpoint activation. (D) quantification of Scells with Mad2GFP on kinetochores.
(2.06 MB EPS)

Figure S2 **Nuclear fission is not affected by the spindle checkpoint.** Dapi staining of cdc11-119 mad2::ura4 cells at 36°C in the presence of either DMSO or MBC.
(2.62 MB EPS)

Figure S3 **No actin structures, other than actin patches, cables, and rings, were observed in cdc11-119 cells treated with 50 µg/ml MBC for 5 h at 36.5°C.** Cells were stained with rodamine phalloidine.
(7.04 MB EPS)

Table S1 **Strains used in this study.**
(0.06 MB DOC)

Video S1 ***cdc11-119 atb2GFP cut11GFP*** **cells grown at 25°C were shifted to 36.5°C for 3 h prior to filming.** Cells were then filmed at 36°C in EMM +DMSO. Frames were taken every 30 s for 20 min using a deltavision microscope under the control of Softworx software.
(3.12 MB MOV)

Video S2 ***cdc11-119 atb2GFP cut11GFP*** **cells were grown at 25°C and then shifted to 36.5°C for 3 h in the presence of 50 µg/ml MBC.** Cells were then filmed at 36°C in EMM +50 µg/ml MBC. Frames were taken every 30 s for 40 min using a deltavision microscope under the control of Softworx software.
(0.65 MB MOV)

Video S3 ***nda3-KM311 uch2GFP*** **cells were grown at 30°C and then shifted to 19°C.** Cells were filmed at 19°C in the presence of 20 µg/ml MBC. Frames were collected every minute using Zeiss Axiovert 200M microscope equipped with a CoolSnap camera (Photometrics) and Uniblitz shutter driver (Photonics, Rochester, NY, USA) under the control of Metamorph software package (Universal Imaging, Sunnyvale, CA, USA).
(0.97 MB MOV)

Video S4 ***cdc11-119 sad1DsRED ndc80GFP*** **cells were grown at 25°C and then shifted to 36.5°C for 3 h in the presence of 50 µg/ml MBC.** Cells were then filmed at 36°C in EMM +50 µg/ml MBC. Frames were taken every 15 s for 10 min using a deltavision microscope under the control of Softworx software. The cell where SPBs and kinetochores divide is in the bottom left corner.
(4.76 MB MOV)

Video S5 ***cdc11-119 cut11mcherry ndc80GFP*** **cells were grown at 25°C and then shifted to 36.5°C for 3 h in the presence of 50 µg/ml MBC.** Cells were then filmed at 36°C in EMM +50 µg/ml MBC. Frames were taken every 15 s for 10 min using a deltavision microscope under the control of Softworx software.
(0.76 MB MOV)

Video S6 ***cdc11-119 ima1::kanMX cut11GFP*** **cells were grown at 25°C and then shifted to 36.5°C for 1 h in the presence of 50 µg/ml MBC.** Cells were then filmed at 36°C in EMM +50 µg/ml MBC. Frames were taken every 30 s for 30 min using a deltavision microscope under the control of Softworx software.
(0.51 MB MOV)

Video S7 ***cdc11-119 cut11GFP*** **cells were grown at 25°C and then shifted to 36.5°C for 3 h in the presence of 50 µg/ml MBC.** LatA was added to the media 10 min prior to filming. Cells were filmed at 36.5°C in EMM +50 µg/ml MBC +10 µM latA. Frames were taken every 30 s for 40 min using a deltavision microscope under the control of Softworx software.
(1.70 MB MOV)

## Abbreviations

LA: Latrunculin A
MBC: carbendazim
SIM: spindle independent mechanism
SPB: spindle pole body
