# Supplementary material for: Fission Yeast Cells Undergo Nuclear Division in the Absence of Spindle Microtubules
Source: PLoS Biol. 2010 Oct 12;8(10):e1000512. doi: 10.1371/journal.pbio.1000512 (PMC2953530; doi:10.1371/journal.pbio.1000512)
Supplement: Table S1 — Strains used in this study. (0.06 MB DOC) [file pbio.1000512.s004.doc]

Supplementary Table 1: Strains used in this study

| Strain | Genotype | Source |
| --- | --- | --- |
| PN1 | 972 h- | Lab collection |
| PN2647 | h- mad2::ura4 ura4-D18 leu1-32 | Lab collection |
| PN1524 | h- cut7-446 leu1-32 | Lab collection |
| PN1502 | h+ cut7-21 ade6- | Lab collection |
| PN15 | h- cdc11-119 | Lab collection |
| PN95 | h- cdc3-124 | Lab collection |
| PN96 | h- cdc4-8 | Lab collection |
| PN98 | h- cdc7-24 | Lab collection |
| PN99 | h+ cdc8-27 | Lab collection |
| PN116 | h- cdc12-112 | Lab collection |
| PN7 | h- cdc25-22 | Lab collection |
| PN113 | h+ cdc11-119 | Lab collection |
| SC339 | h? cdc11-119 atb2GFP:KanMX | Lab collection |
| PN3820 | h? cdc11-119 atb2GFP:kanMX cut11GFP::ura4+ ura4-D18 | Lab collection |
| PN3797 | h+ cdc11-119 cut11-GFP::ura4 ura4-D18 leu1-32 | Lab collection |
| MP59 | h- cdc11-119 cut12-GFP::ura4 ura4-D18 leu1-32 | Lab collection |
| SC220 | h- cdc11-119 sad1RED:leu2 leu1-32 | This study |
| SC299 | h+ cdc11-119 LacO nmt1 promoter-LacI-EGFP-NLS (lys+) leu1-32 ura4-D18 | This study |
| SO1197 | nda3-KM311 ura4-D18 leu1-32 ade6-M2x h- | Oliferenko’s lab |
| SO2243 | nda3-KM311 Uch2p-GFP: ura+ ura4-D18 leu1-32 ade6-M2x h- | Oliferenko’s lab |
| SO2567 | nda3-KM311 Mad2p-GFP: ura+ ura4-D18 leu1-32 ade6-M2x h- | Oliferenko’s lab |
| PN1495 | h- cut11-2 leu1-32 | Lab collection |
| SC281 | cdc11-119 cut11-2 | This study |
| SC280 | cdc11-119 cdc13-117 | This study |
| SC329 | cdc11-119 clp1GFP:ura4 ura4-D18 | This study |
| FY11386 | h- leu1 cut1-645 cdc11-119 | YGRC |
| SC356 | cdc11-119 ndc80GFP:kanMX sad1DsRED:kanMX | This study |
| MKSP22 | h- ima1::kanMX cut11-GFP:ura4+ ura4-D18 leu1-32 | Blobel’s lab |
| SC348 | cdc11-119 ima1:kanMX cut11GFP:ura4 ura4D18 | This study |
| FY15674 | h? ura4-D18 nuf2-2::ura4+ | YGRC |
| SC379 | cdc11-119 nuf2-2:ura4 ura4-D18 | This study |
| SC393 | cdc11-119 cut11-linker-mcherry:ura4 ndc80GFP:kanMX | This study |
| MBY5861 | h- cut11-linker-mcherry:ura4 | Balasubramanian’s lab |
| SC391 | cdc11-119 mad2::ura4 ura4-D18 | This study |
| SC377 | cdc11-119 mad2:GFP-kanMX | This study |
| SC275 | cdc11-119 cut7-446 | This study |
